# Contrast-Enhanced Mammography-Guided Biopsy: Preliminary Results of a Single-Center Retrospective Experience

**DOI:** 10.3390/jcm13040933

**Published:** 2024-02-06

**Authors:** Matteo Sammarra, Claudia Lucia Piccolo, Marina Sarli, Rita Stefanucci, Manuela Tommasiello, Paolo Orsaria, Vittorio Altomare, Bruno Beomonte Zobel

**Affiliations:** 1 Department of Radiology, Fondazione Policlinico Universitario Campus Bio-Medico, 00128 Rome, Italy; m.sammarra@policlinicocampus.it (M.S.); m.sarli@policlinicocampus.it (M.S.); r.stefanucci@policlinicocampus.it (R.S.); m.tommasiello@policlinicocampus.it (M.T.); b.zobel@policlinicocampus.it (B.B.Z.); 2 Department of Breast Surgery, Fondazione Policlinico Universitario Campus Bio-Medico, 00128 Rome, Italy; p.orsaria@policlinicocampus.it; 3 Department of Breast Surgery, Campus Bio-Medico University, 00128 Rome, Italy; v.altomare@policlinicocampus.it; 4 Research Unit of Radiology, Department of Medicine and Surgery, Università Campus Bio-Medico di Roma, Via Alvaro del Portillo, 21, 00128 Rome, Italy

**Keywords:** contrast-enhanced mammography, breast biopsy, breast cancer, contrast media

## Abstract

**Background:** CEM-guided breast biopsy is an advanced diagnostic procedure that takes advantage of the ability of CEM to enhance suspicious breast lesions. The aim pf this paper is to describe a single-center retrospective experience on CEM-guided breast biopsy in terms of procedural features and histological outcomes. **Methods:** 69 patients underwent the procedure. Patient age, breast density, presentation, dimensions, and lesion target enhancement were recorded. All the biopsy procedures were performed using a 7- or 10-gauge (G) vacuum-assisted biopsy needle. The procedural approach (horizontal or vertical) and the decubitus of the patient (lateral or in a sitting position) were noted. **Results:** A total of 69 patients underwent a CEM-guided biopsy. Suspicious lesions presented as mass enhancement in 35% of cases and non-mass enhancement in 65% of cases. The median size of the target lesions was 20 mm. The median procedural time for each biopsy was 10 ± 4 min. The patients were placed in a lateral decubitus position in 52% of cases and seated in 48% of cases. The most common approach was horizontal (57%). The mean AGD was 14.8 mGy. At histology, cancer detection rate was 28% (20/71). **Conclusions:** CEM-guided biopsy was feasible, with high procedure success rates and high tolerance by the patients.

## 1. Introduction

CEM-guided breast biopsy is an advanced diagnostic procedure in the field of breast imaging that exploits the unique features of Contrast-Enhanced Spectral Mammography (CEM) to obtain detailed information on the presence and nature of breast lesions.

CEM is a remarkably promising method that is proposed as a viable alternative to breast MR, especially in terms of cost-effectiveness. The pathophysiological principle on which it is based is similar to MR, namely, it studies tumor neoangiogenesis. This examination allows one to highlight areas of the breast associated with hypervascularized lesions, such as neoplastic proliferations, by intravenous administration of contrast medium.

CEM examinations are performed using a full-field digital mammograph system provided with a Dual-Energy option. After the injection of contrast media, a pair of mammographic images, low energy and high energy, is acquired in rapid succession. The two images are processed using subtraction algorithms with the production of a combined mammographic image (termed “recombined”) to enable the possibility of analyzing the dynamics of enhancement of a suspected lesion, in a similar way to MR [1,2,3].

The main indications for CEM include preoperative staging, inconclusive findings at mammographic and ultrasound imaging, and evaluation of response to neoadjuvant chemotherapy, although these results are extrapolated by retrospective studies [4,5,6].

Magnetic Resonance (MR) is extremely sensitive for the detection of breast cancer. Some malignant lesions are detectable by means of those techniques able to recognize neoangiogenesis, like MR; that is the reason why, to date, MR has been the only option able to sample enhancing-only lesions [6,7,8].

MR-guided vacuum-assisted breast biopsy (VABB) has been demonstrated to be a safe and accurate technique, although it has some drawbacks: it is expensive, not feasible in patients with contraindications, and is not widely available. Moreover, the cancer detection rate as well as false-negative and underestimation rates vary considerably among the published studies [7,8].

Studies have demonstrated that CEM and breast MR have comparable sensitivity in detecting breast cancer. In particular, a leading study by Fallenberg et al. [9], which analyzed the correlation between the two techniques, involved 80 patients affected by breast cancer, with histopathological results as the gold standard. They demonstrated that CEM correlated better with anatomopathological results (Pearson’s correlation coefficient of 0.733) with respect to MR (0.654). A study by Lobbes et al. [6] showed a very high concordance between CEM and MR on tumor size measurements, using surgical specimens as the gold standard, with MR performing slightly better, although the latter suffered from a slight overestimation of measurements, which was not of clinical impact. Van Nijnatten et al. [10] found that the two techniques had comparable results in the assessment of invasive lobular cancer extent, although MR was hindered by more false-positive results. The authors concluded that MR should still be performed for the disease extent in invasive lobular cancers, although CEM might be a valid alternative if breast MR is not available (absolute contraindications, patient suffering from claustrophobia). An important issue when dealing with breast cancer is the evaluation of the contralateral side; Houben et al. [11] evaluated the diagnostic performance of CEM to detect additional lesions in women recalled from screening. In 839 patients, CEM recognized 70 enhancing lesions. Among them, 54.3% were proven to be further foci of cancer, suggesting that CEM could be a feasible technique as a primary staging method, since additional foci of breast cancer can be easily detected, even when mammographically occult or difficult to detect.

When used as a problem-solving tool in cases of inconclusive routinary breast examinations, CEM and breast MR have been shown to have comparable sensitivity. Jochelson et al. [12], in their study involving 52 women undergoing both CEM and breast MR, demonstrated that sensitivity was quite similar between the two techniques (96–100%), with less false-positive findings for CEM than for breast MR. In a multi-reader study with three different readers, Fallenberg et al. [13] evaluated 604 breast lesions (45% were malignant) and concluded that the diagnostic accuracy of CEM was significantly higher than of full-field digital mammography and similar to breast MR. Li et al. [14] analyzed 48 women with breast lesions, studied both with CEM and MR, showing that the two techniques had a sensitivity of 100% for breast cancer detection.

CEM-guided breast biopsy is a relatively new procedure in the field of breast biopsy, which could become a valid alternative to MR-guided breast biopsy in all the cases characterized by neoangiogenesis.

The purpose of this study is to describe a single-center retrospective experience of CEM-guided breast biopsy in terms of the procedural features and histological outcomes of the first cases undergoing this procedure.

## 2. Materials and Methods

### 2.1. Data Collection and CEM Descriptors

A total of 69 CEM-guided breast biopsy procedures were retrospectively analyzed, all performed at the Breast Unit of the Fondazione Policlinico Universitario Campus Bio-Medico in Rome during the period between March 2022 and October 2023. Patients included in the study had a suspicious (BI-RADS 4) or probably malignant (BIRADS 5) finding at contrast-enhanced mammography (CEM). Specifically, in our institution the main indications for CEM include preoperative staging, resolution of problems raised during mammographic and ultrasound screening, evaluation of response to neoadjuvant chemotherapy, and management of lesions of uncertain malignant potential (B3 lesions). Moreover, CEM is the examination of choice in patients with dense breasts and in those who have indications for breast MR in their conventional diagnostic workup but with absolute or relative contraindications to MR (pacemakers or other metallic devices not compatible with MR, claustrophobic patients, patients with a body volume not compatible with MR gantry).

The study was approved by the ethics committee of our hospital, and all patients signed the informed consent.

Exclusion criteria were contraindications to iodinated contrast media.

### 2.2. CEM Protocol

A digital mammography unit (Senographe Pristina, GE Healthcare system) equipped with a specific biopsy add-on unit was used to perform CEM procedures.

Before starting the CEM examination, the patient must be informed about the procedure and possible adverse reactions to the iodinated contrast medium and must provide her consent to the procedure. After an adequate history, including allergic predisposition, as well as assessment of renal function values, a venous access with a 22-G needle was placed in the antecubital fossa. Via an injector, a dose of 1.5 mL/kg of iodinated mdc (300–370 mgI/mL) was administered at a rate of 2–3 mL/s; a bolus of 20 mL of saline was then administered to increase the release of contrast medium into the tissues and improve image quality. After the drug administration was finished, the connecting tube was detached from the patient, while the venous access remained in place until the end of the examination. Image acquisition began two minutes after the injection, striving to finish the examination within 8 min.

During this time, the patient was monitored for any adverse reaction to the iodinated contrast medium. A delay of two minutes after injection is critical since, by beginning breast compression too early, there is a risk that the contrast medium is retained in the vessels outside the breast, preventing it from flowing in the amount needed to be visualized in the early images.

Imaging involved classic CC and MLO projections for both breasts, at low and high energy. Generally, we started with the breast site of the neoplasm in order to be able to highlight early enhancement and reduce false-negative findings from early washout; then, imaging of the contralateral breast was performed. If enhancement was observed in the suspected side, an additional projection was performed after eight minutes to qualitatively assess the kinetics of enhancement and determine the likelihood of malignancy. It is important to emphasize that a small area of enhancement (<5 mm) visible only in the late stage is not considered suspicious but likely attributable to BPE.

Low-energy radiograms were performed with the same kVp as digital mammography, that is, 25–33 kVp, and with the same rhodium or silver filter. High-energy acquisition, on the other hand, was performed with higher kVp values, between 45 and 49, optimizing to the Iodine K-edge and using a copper filter. The copper filter is the best choice because this material is relatively transparent to X-rays at the energies where they are attenuated by iodine, thus providing high contrast in the images. Recombined images were generated by the removal of background glandular tissue and sent to PACS, along with the low-energy images.

Data on patient age, breast parenchyma density, presentation, dimensions, and lesion target enhancement were recorded. In particular, breast density was assessed with the low-energy image according to the American College of Radiology (ACR) BI-RADS^®^ lexicon. The type of enhancement was classified into mass enhancement and non-mass enhancement [15,16,17].

A mass is defined as a space-occupying lesion that displaces tissue.

Morphological descriptors of an enhancing mass lesion include mass shape (round, oval, and irregular), mass margins (circumscribed and non-circumscribed, irregular or spiculated), internal enhancement pattern (homogeneous, heterogeneous, rim enhancement), and the degree of enhancement (subtle, moderate, and intense). The morphological features considered highly suggestive for malignancy were irregular shape, non-circumscribed margins, and heterogeneous internal enhancement; in particular, heterogeneous enhancement appears non-uniform with scattered areas of variable contrast uptake. Moreover, a lesion with moderate or intense enhancement was deemed suspicious of malignant transformation, and, as the literature has demonstrated, most frequently observed in invasive carcinomas (Table 1).

Non-mass enhancement (NME) is defined as an area of enhancement clearly visible in the surrounding parenchyma but without space-occupying features. It may be characterized by scattered areas of glandular tissue or fat within it. It typically refers to an enhancing area different from background parenchymal enhancement, and its most common malignant causes are intraductal or diffuse cancer, particularly invasive lobular carcinoma. It can be focal, linear, segmental, regional, multiregional, or diffuse; specifically, the linear pattern is considered suspicious for malignancy, in particular for DCIS, although it may be the presentation pattern of some lesions of uncertain malignancy potential (B3), such as atypical ductal hyperplasia and lobular carcinoma in situ. Also, the segmental pattern is often observed in neoplastic conditions, representing the involvement of a single branching duct system. The internal enhancement pattern of NME can be classified as a homogeneous, heterogeneous, clumped, or clustered ring. In particular, the clumped enhancement is highly suggestive of malignancy, typically DCIS, as well as the clustered-ring enhancement pattern, which refers to a tiny ring enhancement within an area of heterogeneous NME. The neoplasms most often associated with this pattern are DCIS and invasive cancers associated with ductal carcinoma in situ, maybe because an intraductal cancer with a high degree of neoangiogenesis shows a washout pattern, whereas contrast medium that remains in the periductal stroma demonstrates a persistent and progressive kinetic pattern. A study showed that the specificity of this pattern for malignancy is about 63% [1]. The features considered highly suspicious for malignancy and prone to be sampled were asymmetric NME with a focal, linear, segmental, or regional distribution and a heterogeneous or clumped internal enhancement pattern (Table 2).

In the presence of a suspicious area of NME, the low-energy images were analyzed to search for microcalcifications, which may be associated with the area corresponding to the NME. An important advantage of CEM over MR is the possibility of recognizing breast microcalcifications in the low-energy views and evaluating their morphology and distribution and their conformity to the area of NME in the recombined images.

All the biopsy procedures were performed by means of a 7- or 10-gauge (G) needle.

The procedural approach (horizontal or vertical) and the decubitus of the patient (lateral decubitus or in a sitting position) were noted.

Procedural success was defined by non-visualization of the lesion with enhancement after the biopsy.

Procedural time was recorded, considering the time from the first mammographic image acquired to the scout visualization of clip placement, immediately before breast decompression. In addition, the incidence of any complications (intra-procedural bleeding, vasovagal reactions, allergic reactions, hematomas, or infections) was evaluated.

### 2.3. CEM-Guided Breast Biopsy Procedure

Before performing the procedure, the patient was adequately informed about the risks and benefits, and the patient’s suitability was assessed based on renal function and any allergies to the contrast agent.

The procedure began by choosing the best approach, considering the location of the lesion, by reviewing the previous diagnostic CEM examination, and the physical characteristics of the patient, in order to decide whether to take a medial or lateral approach. The thickness of the compressed breast was used to determine the approach of the biopsy needle, which can be vertical (compressed thickness over 3 cm) or horizontal (compressed thickness 3 cm or less). Medial or lateral approach was performed after calculating the shortest distance from the skin to the target.

The principle of this technique is based on conventional stereotactic guidance, with the addition of the injection of iodinated contrast media at the beginning.

After contrast injection, there is a wait of about 2 min before breast compression, which allows the contrast to be maintained in the lesion for optimal visualization. Moreover, the compression applied during the procedure reduces the washout, and the area of contrast enhancement can be seen for up to 10 min, enough to target the lesion.

When the target is localized, it is compressed by means of a biopsy window, and a pair of low-energy and recombined images are obtained at angles of 0, +15, and −15 degrees. In the same way as for stereotactic-guided biopsy, the needle is pointed toward the target by the machine using a computerized coordination system.

Begore firing, a local anesthesia was administered, and a pre-fire imaging of low-energy and recombined views was obtained in order to evaluate whether the target remained in the correct position. Then, after confirming the correct positioning of the biopsy needle, it was fired through the target, and multidirectional samplings in a complete clockwise rotation were performed.

**Figure 1:**
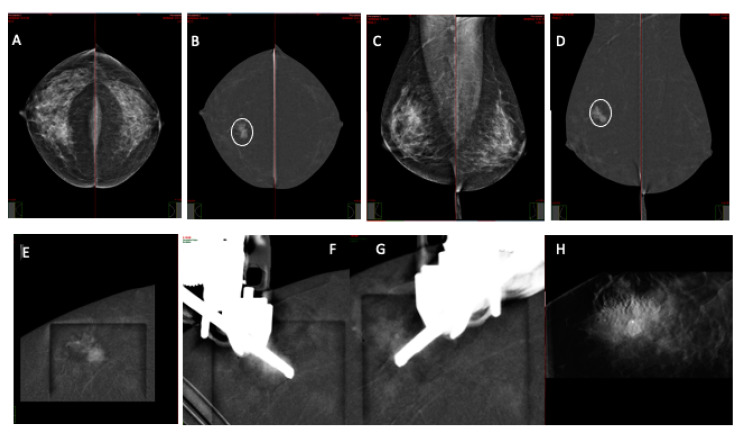
An asymptomatic 49 yo patient was referred for a CEM examination after a routine external center examination. Low-energy CEM CC view (**A**) and MLO (**C**) show an irregular area of higher density in the outer inner quadrant of the right breast, appearing as a 25 mm enhancing mass (circle) on recombined images (**B**,**D**). Right breast CEM-guided biopsy was performed (**E**–**H**) with horizontal needle approach. Scout-view imaging at 0 degrees (**E**). Pre-fire imaging with stereotactic pair at −15 and 15 degrees (**F**,**G**). Post-biopsy with marker placement (**H**). Pathology report: invasive ductal cancer NOS, G1, pT 1b pN 0.

**Figure 2:**
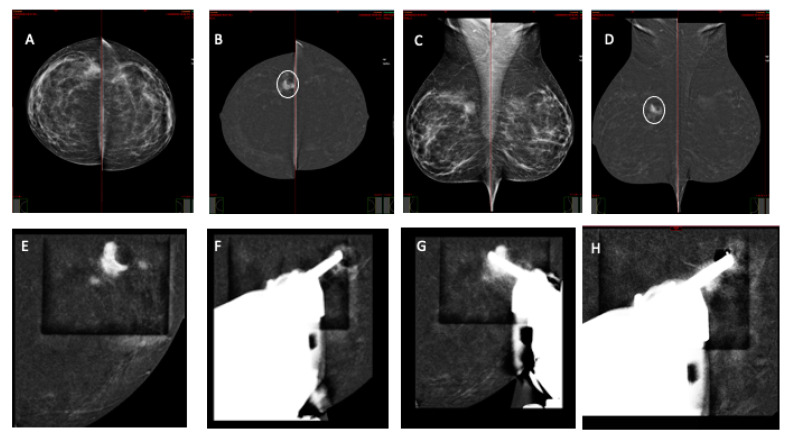
An asymptomatic 45 yo patient was referred for a CEM examination after a suspicious finding in a routine external center examination. Low-energy CEM CC view (**A**) and MLO (**C**) show an irregular mass in the upper outer quadrant of the right breast, appearing as a 20 mm enhancing mass (circle) with satellite nodules at the periphery of a round area of radiolucency with regular margins on recombined images (**B**,**D**). Right breast CEM-guided biopsy was performed (**E**–**H**) with vertical needle approach with patient in sitting position. Scout-view imaging at 0 degrees (**E**). Pre-fire imaging with stereotactic pair at −15 and 15 degrees (**F**,**G**). Post-biopsy with marker placement (**H**). Pathology report: invasive ductal cancer NOS, G2.

Once the biopsy was completed, a stereotactic marker was placed to localize the lesion in the future (Figure 1 and Figure 2).

## 3. Results

A total of 69 patients underwent a CEM-guided biopsy, with 2 of them showing two synchronous lesions in both breasts, while in 1 patient the procedure was not performed because the suspicious contrast enhancement area was not detected in the procedural examination.

The median age was 52 years; breast density was classified as dense in 82% and non-dense in 18%. The suspicious lesions, which showed contrast enhancement in CEM just before performing the breast biopsy, presented as mass enhancement in 35% of cases and non-mass enhancement in 65% of cases. The median size of the target lesions was about 20 mm.

The procedure success rate was 97.1%; in two patients, the target lesion was partially masked by a severe background parenchymal enhancement, so a digital breast tomosynthesis (DBT) acquisition was performed in order to carry out a DBT-guided biopsy, resulting in an invasive ductal carcinoma NST.

The median procedure time for each biopsy was 10 ± 4 min. The patients were placed in a lateral decubitus position in 52% of cases and seated in 48% of cases. The most common approach used was horizontal (57%). The mean AGD was 14.8 mGy.

Early complications were hematomas in 20 patients (29%) and vasovagal reactions in 4 (5.7%).

As regards the pathological results, the cancer detection rate was 28% (20/71); among these, there were 3 DCIS and 17 invasive cancers. Among non-neoplastic lesions, 3 (4.2%) were B1, 24 (33.8%) were B2 (mainly sclerosing adenosis and fibrocystic mastopathy), and 24 (33.8%) were B3 (mainly LIN and ADH).

The patients with biopsy-proven cancer underwent surgery, which confirmed the biopsy results.

Patients with a benign diagnosis were followed up on a regular basis.

Adverse reactions to the contrast medium were not observed, mainly because every patient underwent a careful analysis of any possible previous allergy to iodinated contrast media. The demographic and procedural data are summarized in Table 3.

## 4. Discussion

CEM-guided biopsy is a relatively new technique that may help in the characterization of suspicious enhancing breast lesions, as a valid alternative to MR.

MR-guided biopsy is performed when an area of suspicious contrast enhancement, not detectable by means of mammographic and ultrasound imaging, is evident on post-contrast images. This is a feasible technique which is safe and is not hindered by the drawback of ionizing radiation. Nevertheless, it has some limitations related to the localization of the target, which can last very long, with an imaging time of 35–41 min and whole examination time of around 60–70 min [18,19]. This because the choice of the best approach for the biopsy requires a careful review of the diagnostic MR images to understand the site, depth, distance from the nipple, and the two-view visualization. Other drawbacks are the high costs, the limited availability, and the expertise of the clinician; in fact, successful MR-guided biopsies need skilled, experienced radiologists and technologists who are dedicated to breast imaging and breast biopsy and can problem-solve when faced with cases that require additional prebiopsy planning [20].

Previous studies about MR-guided biopsy success rate reported values ranging from 87 to 98%; in our series, the biopsy success rate was 97.1%. In two patients, the target lesion was partially masked by a severe background parenchymal enhancement, so a digital breast tomosynthesis (DBT) acquisition was performed in order to carry out a DBT-guided biopsy, resulting in an invasive ductal carcinoma NST. A possible explanation of this finding could be related to a focal area of background parenchymal enhancement secondary to noncyclical hormonal factors or maybe to fibrocystic changes. The non-visualization of a previously detected suspicious lesion has been reported in 8–13% of MR-guided biopsies [21,22,23].

In our series, the cancer detection rate was 28% (20/71), which is in line with results obtained by means of MR-guided breast biopsy, ranging from 18 to 61%. This is a critical point when dealing with MR-guided biopsy, because there are some drawbacks related to the inherent uncertainties in the accuracy of sampling; in addition, the radiography of the samples is not available, as in the case of MR-guided biopsies, and the biopsy needle cannot be monitored in real time as with ultrasound-guided biopsies.

The CEM procedure involves doses of ionizing radiation. It is important to discuss cost, radiation, and potential risks with the patient, including allergic reactions to the contrast medium and the risk of renal failure related to iodine use. Careful risk management and clear communication are essential to ensure the safety and efficacy of the procedure.

To date, there are only a few papers published on this topic, showing promising results on this technique [24,25,26].

In our study, the procedure was correctly performed in all cases, with a 100% success rate. The approach most often chosen was horizontal, which is safer in patients with small breasts, but with a slight increase in the time of the procedure, and the position most often used was the lateral decubitus, which helped in reducing anxiety in the patients.

Few data are available about the AGD, since radiation exposure is a major drawback of CEM-guided biopsy.

Alcantara et al. [25] reported a low median number of scout views before targeting, avoiding additional image acquisitions after tissue sampling. Cheung et al. [24] obtained an AGD of 14.3 ± 12.3 mGy; they used the recombined image to evaluate target location and then marked the skin before biopsy [6].

In the present study, the mean AGD was 14.8 ± 10.2 mGy, which is in line with Cheung et al.’s results [24]. We centered the target chosen on the diagnostic CEM, marked the skin, and then administered the contrast medium. In this way, we correctly localized all the target lesions. The mean ADG in our series was lower than that of stereotactic biopsy (about 22 mGy) but higher than that of DBT (about 10 mGy).

The most common complications were hematomas and vasovagal reactions, in line with data from the literature, that is, 1–5% for vasovagal reactions and 2–83% for hematomas, which are common events, but with a low clinical impact. The MR-guided VAB is characterized by a comparable complication rate of VAB under stereotactic guidance, despite higher technical requirements, in particular hemorrhages. The complication rate of this procedure lies within the well-established range of complication rates (2–14%) [27,28].

In a review which compared the technical performance of MR-guided biopsy and stereotactic-guided and ultrasound-guided techniques, involving 9113 VAB procedures, the authors reported that there were no cases of bleeding requiring surgical intervention, so it could be defined as a safe technique [19].

No severe allergic reactions were observed, mainly because every patient underwent a careful analysis of any possible previous allergy to iodinated contrast media.

The main limitation of the current study is the small sample size, which enabled us to assess the accuracy of the technique as well as the data about AGD. Nevertheless, the AGD per exposure was always under the threshold of 3 mGy, set by the Mammography Quality Standards Act regulations. This issue requires further investigation.

## 5. Conclusions

In conclusion, this study showed that this technique is well tolerated by patients, is feasible because it is performed in a short time with high rates of success, and should be considered as a promising alternative to MR breast biopsy. Nevertheless, several studies are needed to demonstrate its application on a vast scale.

## Figures and Tables

**Table 1:** Mass descriptors of malignant lesions.

Shape	Margins	Internal Enhancement Pattern	Contrast Enhancement
Irregular	Non-circumscribed	Heterogeneous	Moderate
		Ring-enhancement	Intense

**Table 2:** Non-mass descriptors of malignant lesions.

Spatial Distribution	Internal Enhancement Pattern	Symmetric/Not Symmetric
Linear	Heterogeneous	Not symmetric
Segmental	Clumped	
	Clustered-ring	

**Table 3:** Demographic and procedural data of CEM-guided biopsy.

Variables	Value (*n* = 69)
**Age (mean, range), yrs**	52 (range: 45–77)
**Breast density,** ***n* %**	
Dense	57 (82%)
Non-dense	12 (18%)
**Imaging findings, %**	
Mass	24 (35%)
Non-mass	45 (65%)
**Procedural time (mean ± SD), min**	10 ± 4 min
**Needle approach, *n* (%)**	
Vertical	12 (21%)
Horizontal	57 (79%)
**AGD (mean ± SD)**	14.8 ± 10.2
**Biopsy results**	
B2 *n* (%)	24 (33.8%)
B3 *n* (%)	24 (33.8%)
Tumors, *n* (%)	20 (28%)

## Data Availability

Personal data is unavailable due to privacy restrictions.
